# A Clinicopathological Description of Kidney Features in VEXAS Syndrome

**DOI:** 10.1016/j.ekir.2024.10.026

**Published:** 2024-10-28

**Authors:** Martin Mathurin, Pierre Hirsch, Vincent Jachiet, Jérôme Hadjadj, Guillaume Le Guenno, Mael Heiblig, Solenne Pelletier, Alexis Mathian, Cyril Garrouste, Aurélie Lavergne, Samuel Ardois, Joanne Flejeo, Ingrid Masson, Zakaria Boukerroucha, François Perrin, Marion Magnol, Arnaud Constantin, Jérémie Dion, Thibault Comont, Antoine Huart, Jean-Louis Kemeny, Cécile Picard, Magali Colombat, Cécile Le Naoures, Laurent Benard, Aurélie Sannier, François Vrtovsnik, Isabelle Brocheriou, Cedric Pastoret, Pierre Sujobert, Eric Delabesse, Olivier Kosmider, François Delhommeau, Arsène Mekinian, Sophie Georgin-Lavialle, David Buob, Khalil El Karoui, FRENVEX Group

**Affiliations:** 1 Médecine Interne, Hôpital Tenon, Sorbonne Université, Paris, France; 2 Sorbonne Université, INSERM, Centre de Recherche Saint-Antoine, CRSA, AP-HP, Siric Curamus, Paris, France; 3 Hôpital Saint-Antoine, Service d'Hématologie Biologique, Sorbonne Université, Paris, France; 4 Médecine Interne, Hôpital Saint-Antoine, Sorbonne Université, Paris, France; 5 Médecine Interne, Hôpital Estaing, Clermont-Ferrand, France; 6 Hématologie, Hôpital Lyon Sud-HCL, Lyon, France; 7 Néphrologie, CHU Lyon-HCL, Lyon, France; 8 Médecine Interne 2, Hôpital Pitié-Salpétrière, Paris, France; 9 Néphrologie, Hôpital Gabriel Montpied, Clermont-Ferrand, France; 10 Néphrologie, CHU de Rennes, Rennes, France; 11 Médecine Interne, CHU de Rennes, Rennes, France; 12 Néphrologie, GHT d’Armor, Saint-Brieuc, France; 13 Néphrologie, Centre Hospitalier de St Etienne, France; 14 Néphrologie, Centre Hospitalier de Saint-Nazaire, Saint-Nazaire, France; 15 Médecine Interne, Centre Hospitalier de Saint-Nazaire, Saint-Nazaire, France; 16 Médecine Interne, IUCT-Oncopole, Toulouse, France; 17 Néphrologie, Hôpital de Rangueil, Toulouse, France; 18 Anatomopathologie, Hôpital Gabriel Montpied, Clermont-Ferrand, France; 19 Anatomopathologie, Hospices Civils de Lyon, Lyon, France; 20 Anatomopathologie, CHU de Toulouse, Toulouse, France; 21 Anatomopathologie, CHU de Rennes, Rennes, France; 22 Anatomopathologie, Centre Hospitalier de Saint-Nazaire, Saint-Nazaire, France; 23 Anatomopathologie, Hôpital Bichat - Claude-Bernard, Paris, France; 24 Néphrologie, Hôpital Bichat - Claude-Bernard, Paris, France; 25 Anatomopathologie, Hôpital Pitié-Salpêtrière, Paris, France; 26 Hématologie - Biologie Moléculaire, CHU Pontchaillou, Rennes, France; 27 Hématologie Biologique, Hospices Civils de Lyon, Hôpital Lyon Sud, Lyon, France; 28 Hématologie Biologique, Institut Universitaire de Cancérologie de Toulouse site Oncopôle, Toulouse, France; 29 Hématologie Biologique, Institut Cochin, Paris, France; 30 Anatomopathologie, Hôpital Tenon, Sorbonne Université, Paris, France; 31 Néphrologie, Hôpital Tenon, Sorbonne Université, Paris, France

**Keywords:** acute kidney injury, chronic kidney disease, inflammation, kidney biopsy, UBA1, VEXAS

## Introduction

VEXAS (Vacuoles, enzyme E3, X-linked, Autoinflammatory, Somatic) is a recently reported late-onset autoinflammatory syndrome characterized by myeloid lineage-restricted somatic mutations in the ubiquitin-activating E1 (UBA1) gene.^1^ The estimated prevalence of disease-causing *UBA1* variants was 1 in 4269 men aged >50 years in 163.096 exome data from a US regional health system.^2^ The main clinical features of VEXAS include skin lesions (83%, mostly neutrophilic dermatosis), fever (64%), lung involvement (50%), and ocular inflammation (39%).^1^^,^^3^ Myelodysplastic syndrome is identified in about 50% cases. Interestingly, direct organ infiltration by immature clonal cells could have a critical role in disease pathophysiology.^4^^,^^5^ To date, renal lesions of patients with VEXAS have been poorly characterized, on the basis of single case reports only, and include interstitial nephritis, AA amyloidosis, or pauci-immune vasculitis.^6^, ^7^, ^8^, ^9^ In the present work, we aimed to describe the clinico-pathological findings and molecular characterization of patients with VEXAS who underwent a kidney biopsy.

## Results

Among 303 patients with VEXAS with somatic pathogenic UBA1 variants, we identified 11 patients with kidney biopsy. Three patients were reported on previously.^6^^,^^7^^,^^9^ Renal biopsy was performed between 2012 and 2023 (with retrospective molecular diagnosis in 8 patients). Clinical characteristics of renal biopsy are presented in Table 1. All patients were male, mean (minimum–maximum) age was 70 (59–78) years, hemoglobin was 8.77 (6.2–11) g/dl, mean corpuscular volume was 104 (95–118) μ^3^, and C-reactive protein was elevated in all patients. Renal presentation included progressive chronic kidney disease in 5 patients and acute kidney injury in 6, including two patients (patients 7 and 9) needing renal replacement therapy. All patients presented with extrarenal manifestations (Table 1). Interestingly, acute kidney injury episodes were chronologically linked with VEXAS flares in 3 patients (patients 2, 3, and 11). Most patients (*n* = 10/11, 90%) were already exposed to steroids or immunomodulators at renal biopsy. Nephrotic range proteinuria was observed in 5 patients. Mean serum creatinine was 363 μmol/l; leukocyturia and hematuria were present in 5 and 6 patients respectively. Seven patients presented with an associated myelodysplastic syndrome. Monoclonal gammopathy of undetermined significance was identified in 6 patients, with IgM circulating clones in 5 of 6 patients. Renal function evolution was usually favorable after treatment with corticosteroids and various immunosuppressants, despite frequent infectious complications (Supplementary Table S1).

**Table 1:** Clinical and biological characteristics at kidney biopsy

Patient^a^	Age	AKI	CKD	Systemic symptoms	Treatment at KB	sCreatinine (μmol/l)	eGFR (ml/min per 1.73 m^2^)	Albuminemia (g/l)	Pu (g/d)	Albuminuria (% of proteinuria)	Hematuria	Leucocyturia	Hb (g/dl)	MCV (μ^3^)	CRP (mg/l)	Monoclonal gammopathy	Associated MDS	Autoimmunity
1	61	0	1	HLA B27+, U, IBD, RP, S (ND), sclerosing panniculitis, possible SS	Steroids 30mg/d + Aza	70	110	NA	0.12	NA	+	NA	8.9	108	75	IgM Kappa	Yes	Type II cryoglobulinemia
2	72	1	0	L, S (vasculitis), IBD, RP	0	1300	0	2.1	1.8	NA	+	+	6.2	97	291	0	Yes	AAN 1/320
3	69	1	0	A, S, IBD, IS, CI, L, SS, RP, E	Hydro-cortisone 30mg/j	169	35	3.5	0.7	22%	+	0	10.4	99	37	0	NA	AAN 1/640
4	69	0	0	E, A, VTE, S(ND), orchitis, L	Steroids 20mg/d	72	90	2.3	5.6	NA	+	0	8.5	102	160	0	Yes	0
5	73	1	0	A, S (ND), IBD	Steroids 25mg/d	980	NA	2.5	6.3	NA	+	+	9.0	118	137	IgM kappa and IgM lambda	Yes	0
6	67	0	0	E, RP, S(vasculitis)	Steroids 50mg/d	43	113	NA	3.0	NA	NA	+	6.6	NA	175	IgM lambda	Yes	NA
7	73	1	1	S (vasculitis), A, U	Steroids 50mg/d	296	NA	3.4	1.2	NA	0	+	8.7	95	92	IgM lambda, IgG lambda	Yes	0
8	77	0	1	S (ND), A, RP	Steroids 3mg/d (+ Upada-citinib)	191	29	NA	4.3	72%	0	+	11.0	NA	37	0	Yes	NA
9	73	1	1	S(ND), peripheral neuropathy, meningitis, VTE	Steroids <10mg/d + Ruxo	239	22	NA	0.3	NA	0	0	9.1	102	26	IgG lambda	NA	NA
10	59	0	1	A, S, RP	Steroids	427	12	NA	9.0	NA	NA	NA	8.13	107	97	NA	NA	NA
11	78	1	0	S (vasculitis + ND), A,	Steroids 7mg/d	207	26	NA	3.8	NA	+	NA	9.90	NA	237	IgM Kappa	No	0

A, arthritis; AKI, acute kidney injury; AS, ankylosing spondylitis; Aza, azathioprine; CI, cardiac infarction; CKD, chronic kidney disease; CRP, C-reactive protein; E, episcleritis; Hb, hemoglobin; Hu, hematuria; IBD, intestinal bowel disease; IS, ischemic stroke; KB, kidney biopsy; L, lung involvement; Lu, leukocyturia; MCV, mean corpuscular volume; MDS, myelodysplastic syndrome; NA, non available; ND, neutrophilic dermatosis; NS, nephrotic syndrome; Pu, proteinuria; RP, relapsing polychondritis; Ruxo, ruxolitinib; S, skin; SS, Sicca syndrome; U, uveitis; VTE, venous thromboembolism.

a Patients 1, 2 and 10 were previously reported on.^6^^,^^7^^,^^9^

**Figure 1:**
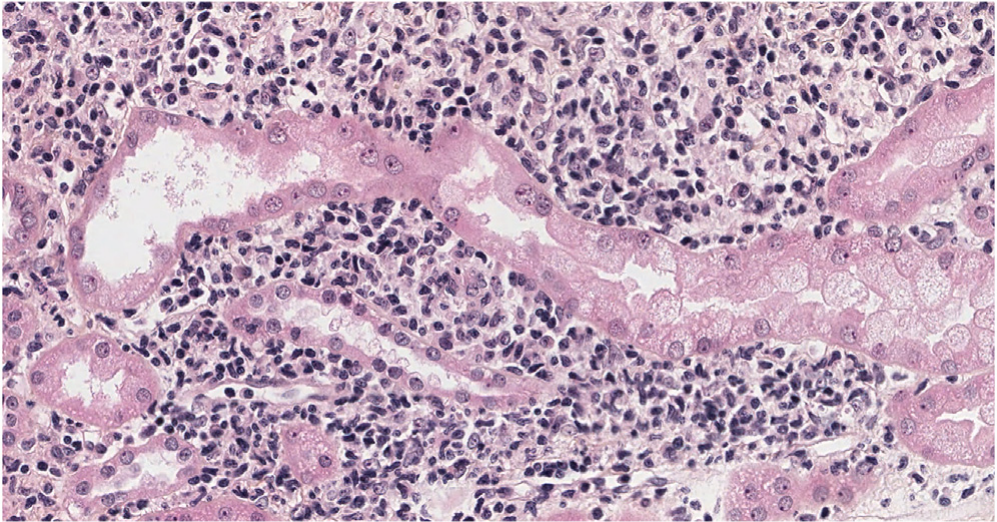
Renal biopsy: severe interstitial inflammatory infiltration with presence of lymphocytes and plasma cells, admixed with macrophages and neutrophil cells. (HES, ×20).

Histopathological findings are depicted in Supplementary Tables S2 and S3. Primary diagnoses included acute interstitial nephritis (*n* = 4), antineutrophil cytoplasmic autoantibody negative pauci-immune vasculitis (*n* = 1), IgA nephropathy superimposed with minimal change disease (*n* = 1), IgA nephropathy (*n* = 1), diabetic nephropathy with extensive fibrosis (*n* = 1), acute tubular necrosis (*n* = 2), and AA amyloidosis (*n* = 1). No patients had monotypic deposits. Interestingly, interstitial inflammatory infiltration was noticed in 8 of 11 cases, whatever the primary diagnosis (Figure 1). Immunohistochemical characterization of interstitial inflammatory cells demonstrated the presence of lymphocytes and plasma cells, admixed with macrophages and neutrophil cells. Myeloperoxidase and CD68 immunostaining was positive in all cases, with a variable degree of expression (Supplementary Table S2 and Figure S1). Notably, 4 cases showed infiltrative cells expressing both myeloperoxidase and CD68, suggesting the presence of immature clonal cells of the myeloid lineage. In line with this observation, to identify the presence of UBA1-mutated clone in kidney samples, we performed molecular analysis of DNA extracted from the 8 available frozen samples by next generation sequencing using a panel of 65 genes implied in myeloid malignancies (including UBA1). UBA1 variation was detected in 7 of 8 samples, with variant allele frequency ranging from 2% to 17% of total kidney DNA (Supplementary Table S2), with other associated clones in only 2 of 8 kidney samples (Supplementary Table S2).

## Discussion

In this work, we report the first series focusing on clinicopathological and molecular renal features of patients with VEXAS. We show that clinical and histological presentation is diverse, and that UBA1 clone is detected within the kidney in most cases (88%).

The number of patients who experienced renal biopsy among the large cohort of the French VEXAS network (11/303 patients, ∼3.6%) may suggest that renal disease associated with VEXAS is rare. However, renal injury is probably underdiagnosed in this population with a high risk of postbiopsy complications, due to altered general condition and frequent thrombocytopenia. In addition, the improvement of renal dysfunction after steroid increase in some patients may also have limited kidney biopsy indications.

The different clinical and histological presentations (acute interstitial nephritis, pauci-immune vasculitis, IgA-dependent diseases, minimum change disease, or AA amyloidosis) are in line with previous single case reports, or diagnoses observed in myelodysplastic syndrome contextS1 and other autoinflammatory diseases.S2 Notably, interstitial infiltration appears as a frequent hallmark of renal biopsies of patients with VEXAS, despite frequent treatment with various immunomodulators. Moreover, the favorable evolution of acute kidney injury after steroid treatment suggests that renal disease could represent a manifestation of VEXAS flare. Our systematic analysis of all renal biopsies within the French VEXAS cohort also reveals diagnoses with no direct link with VEXAS, such as postsepsis acute tubular necrosis or diabetic nephropathy. Consequently, histological analysis appears essential to characterize renal involvement in elderly men with autoinflammatory symptoms suggestive of VEXAS syndrome.

In this work, we provide a molecular analysis of clones of the myeloid lineage (including UBA1) in renal biopsy, suggesting the frequent presence of UBA1 clones within the kidney. The variant allele frequency of the UBA1 clones (until 17% of whole kidney DNA) suggests the presence of clonal cells within the kidney, rather than a nonspecific contamination by circulating clonal cells. Thus, UBA1 clones could infiltrate solid organ tissues (such as kidney or skin, as previously reported^5^), although no definitive conclusion regarding their pathophysiological role could be drawn from this small, retrospective cohort. Interestingly, kidney infiltrates frequently showed both myeloperoxidase and CD68 expression suggestive of pathogenic clonal cells, as previously described in neutrophilic dermatoses associated with VEXAS.^5^ This supports a similar mechanism of clonal infiltration leading to kidney and skin involvement. However, the demonstration of the pathophysiological impact of *UBA1* clones in kidney injury would need specific experiments in a murine model bearing myeloid *UBA1* variant, as previously shown for clonal hematopoiesis of indeterminate potential.S3 Interestingly, *UBA1* clones were even detected within kidney samples with extensive inflammatory fibrosis, suggesting that the proinflammatory clones may also favor fibrosis induction, as previously reported.S3^,^S4

The limitations of this study are inherent in its retrospective nature. The low number of patients limits the generalizability of these data. Similarly, the absence of concomitant kidney and blood evaluation of UBA1 variant allele frequency prevents the comparison of kidney and peripheral variant allele frequency. However, our detailed pathological, immunohistochemical, and molecular data provide a unique characterization of kidney lesions in VEXAS syndrome.

In conclusion, this series highlights the heterogeneous clinical and histological presentation of VEXAS-associated kidney disease. The molecular analysis of kidney tissue along with the frequent histological finding of interstitial infiltration may suggest a specific involvement of clonal cells directly targeting the kidney during VEXAS, thereby expanding the characterization of renal diseases associated with myeloid clones.

## Appendix

### List of the FRENVEX Group

Jérome Hadjadj, Yann Nguyen, Rim Bourguiba, Mael Heiblig, Chloé Mc Avoy, Valentin Lacombe, Samuel Ardois, Thibault Comont, Estibaliz Lazaro, Francois Lifermann, Guillaume Le Guenno, Herve Lobbes, Vincent Grobost, Roderau Outh, Yvan Jamilloux, Alexis Mathian, Jonathan Broner, Thomas Moulinet, Achille Aouba, François Chasset, Laurent Arnaud, Pierre Sujobert, Pierre Hirsch, Noémie Abisror, Pierre Fenaux, Olivier Kosmider, Vincent Jachiet, Olivier Fain, Benjamin Terrier, Sophie Georgin-Lavialle, and Arsène Mekinian.

## Disclosure

All the authors declared no competing interests.

## Data Availability Statement

The data in this study were extracted from confidential medical records. French legislation does not authorize their free use and transfer. This data cannot be made freely available. Data may be shared as part of a collaborative project, subject to further validation by an institutional review board and patient consent.

## Supplementary Material

Supplementary File (PDF) **Supplementary Methods. Supplementary References. Figure S1.** Representative CD68 (A 40X, C 20X) and MPO (B 40X, D 20X) immunostaining. **Table S1.** Evolution at last follow-up. **Table S2.** Histological, immunohistochemical and molecular characteristics. **Table S3.** Detailed histological description. STROBE Statement.

## Footnotes

Supplementary File (PDF)

**Supplementary Methods.**
**S****upplementary References**. **Figure S1.** Representative CD68 (A 40X, C 20X) and MPO (B 40X, D 20X) immunostaining. **Table S1.** Evolution at last follow-up. **Table S2.** Histological, immunohistochemical and molecular characteristics. **Table S3.** Detailed histological description. STROBE Statement.
