# Supplementary material for: A Clinicopathological Description of Kidney Features in VEXAS Syndrome
Source: Kidney Int Rep. 2024 Oct 28;10(1):260–4. doi: 10.1016/j.ekir.2024.10.026 (PMC11725828; doi:10.1016/j.ekir.2024.10.026)
Supplement: Supplementary File (PDF) — Supplementary Methods. Supplementary References. Figure S1. Representative CD68 (A 40X, C 20X) and MPO (B 40X, D 20X) immunostaining. Table S1. Evolution at last follow-up. Table S2. Histological, immunohistochemical and molecular characteristics. Table S3. Detailed histological description. STROBE Statement. [file mmc1.pdf]

Supplementary Methods

1. Patient cohort

Patients included in this cohort fulfill the following inclusion criteria:

- VEXAS syndrome with compatible clinical presentation and confirmed somatic pathogenic *UBA1* variant

- Adult patients with kidney involvement and renal biopsy performed at the local center

Patients 'records have been reviewed and relevant clinical and biological data, including clinical symptoms, renal function (evaluated using the CKD-EPI formula), proteinuria, hematuria, leucocyturia, hemogram, and inflammatory syndrome have been extracted.

2. Renal pathology

Kidney biopsy samples have been processed for light microscopy and immunofluorescence following routine techniques. A detailed, centralized review (Pr D. Buob, Department of Pathology, Tenon Hospital, Sorbonne University) was performed. Vascular lesions, tubular atrophy, interstitial fibrosis, and cellular infiltration were graded according to the Banff classification (ref S5). Cell type identification and characterization used the following immunohistochemical markers on AFA-fixed paraffin embedded samples: CD3 (T lymphocyte marker), CD68 (macrophagic marker), CD15 (mature neutrophils marker), MPO (neutrophil lineage marker).

3. Molecular evaluation

Sanger analysis performed on DNA extracted from circulating leukocytes was used to detect *UBA1* pathogenic variants on a routine basis in all patients. Detected *UBA1* mutation is reported in Table 2. Regarding kidney sample analyses, DNA was extracted from frozen kidney samples using Qiagen kit according to manufacturer instructions. For a majority of samples, libraries were obtained from 200 ng of DNA, using custom myeloid solution (Sophia genetics) and according to the manufacturer protocol. Sequencing was performed using a Miseq sequencer (Illumina). Alignments and variant calling was performed by a dedicated bioinformatics pipeline using Sophia DDM software (Sophia genetics), using a panel of 65 genes implied in myeloid malignancies (including *UBA1*). The sensitivity was 1%. Depth of coverage was at least 500x in more than 90% target genes (including *UBA1*). All variants were checked using Integrative Genomics Viewer (IGV) software v2.3. Variant allele frequency was defined as the frequency of detected variant to total DNA analyzed.

We searched for patients with renal biopsy among 303 VEXAS cases from the French national VEXAS cohort. We extracted relevant data from medical records, and performed a centralized review of renal biopsies, including an immunohistochemical characterization of renal infiltrate. Moreover, we investigated the presence of UBA1 mutated clones within the kidney by sequencing DNA extracted from whole kidney tissue.

This study was conducted in compliance with the Good Clinical Practices protocol and Declaration of Helsinki principles and received approval from the Cochin Hospital Institutional Review Board (CLEP Decision N°: AAA-2021-08040).

Supplementary references

S1. Schwotzer N, Provot F, Ville S, Daniel L, Le Fur A, Kissling S, et al. Spectrum of Kidney Involvement in Patients with Myelodysplastic Syndromes. Kidney Int Rep. 2021 Mar;6(3):746–54.
S2. Vaglio A, Gattorno M, McAdoo S, Obici LP, Ghiggeri GM. Editorial: The kidney in auto-immune and auto-inflammatory processes: Definitions, mechanisms, and biomarkers. Front Med. 2022;9:1129021.
S3. Vlasschaert C, Robinson-Cohen C, Chen J, Akwo E, Parker AC, Silver SA, et al. Clonal hematopoiesis of indeterminate potential is associated with acute kidney injury. Nat Med. 2024 Mar 7;
S4. Jaiswal S, Natarajan P, Silver AJ, Gibson CJ, Bick AG, Shvartz E, et al. Clonal Hematopoiesis and Risk of Atherosclerotic Cardiovascular Disease. N Engl J Med. 2017 Jul 13;377(2):111–21.
S5. Loupy A, Haas M, Roufosse C, et al. The Banff 2019 Kidney Meeting Report (I): Updates on and clarification of criteria for T cell– and antibody-mediated rejection. Am J Transplant. 2020;20:2318–2331.

List of the 65 analysed genes :

ANKRD26 (NM_014915.3), ASXL1 (NM_015338.6), ASXL2 (NM_018263.6), ATM (NM_000051.4), BCOR (NM_001123385.2), BCORL1 (NM_001184772.2), BRAF (NM_004333.6 ; ex.11 ;15), CALR (NM_004343.4 ; ex.8-9), CBL (NM_005188.4), CCND3 (NM_001760.5), CEBPA (NM_004364.4), CSF3R (NM_000760.4), CSNK1A1 (CSNK1A1 (NM_001892.5), DDX41(NM_016222.4), DNMT3A (NM_022552.4), EIF6 (NM_002212.3), EP300 (NM_001429.4), EPOR (NM_000121.4), ETNK1(NM_018638.5), ETV6 (NM_001987.5), EZH2 (NM_004456.5), FLT3 (NM_004119.3), GATA1 (NM_002049.4), GATA2 (NM_032638.5), HRAS (NM_5343.3 ex 2-4), IDH1 (NM_005896.3 ex6), IDH2 (NM_002168.4 ex 4), JAK2 (NM_004972.4), KIT (NM_000222.3), KRAS (NM_004985.5 ex 2-4), MPL (NM_005373.3), NF1 (NM_001042492.3), NFE2 (NM_001136023.2), NPM1 (NM_002520.7 ex 11), NRAS (NM_002524.5 ex 2-3), PHF6 (NM_001015877.2), PPM1D (NM_003620.4), PRPF8 (NM_006445.4), PTPN11 (NM_002834.5), RAD21 (NM_006265.3), RBBP6 (NM_6910.5), RPL23 (NM_000978.4), RUNX1 (NM_001754.5), SBDS (NM_016038.4), SETBP1(NM_015559.3), SF3B1 (NM_012433.4), SH2B3 (NM_005475.3), SMC1A (NM_6306.4), SMC3 (NM_5445.4), SRP68 (NM_014230.4), SRP72 (NM_006947.4), SRSF2 (NM_001195427.1), STAG1 (NM_005862.3), STAG2 (NM_001042750.1), TERC (NR_001566.1), TERT (NM_198253.3 : -57 -124 ;-146), TET2 (NM_001127208.3), TET3 (NM_001287491.2), THPO (NM_000460.4), TP53 (NM_000546.5), U2AF1 (NM_006758.2), U2AF2 (NM_007279.3), UBA1 (NM_3334.4 ex 3), WT1 (NM_024426.6), ZRSR2 (NM_5089.4), SNP en 14q32 (dup ATG2B/GSKIP).

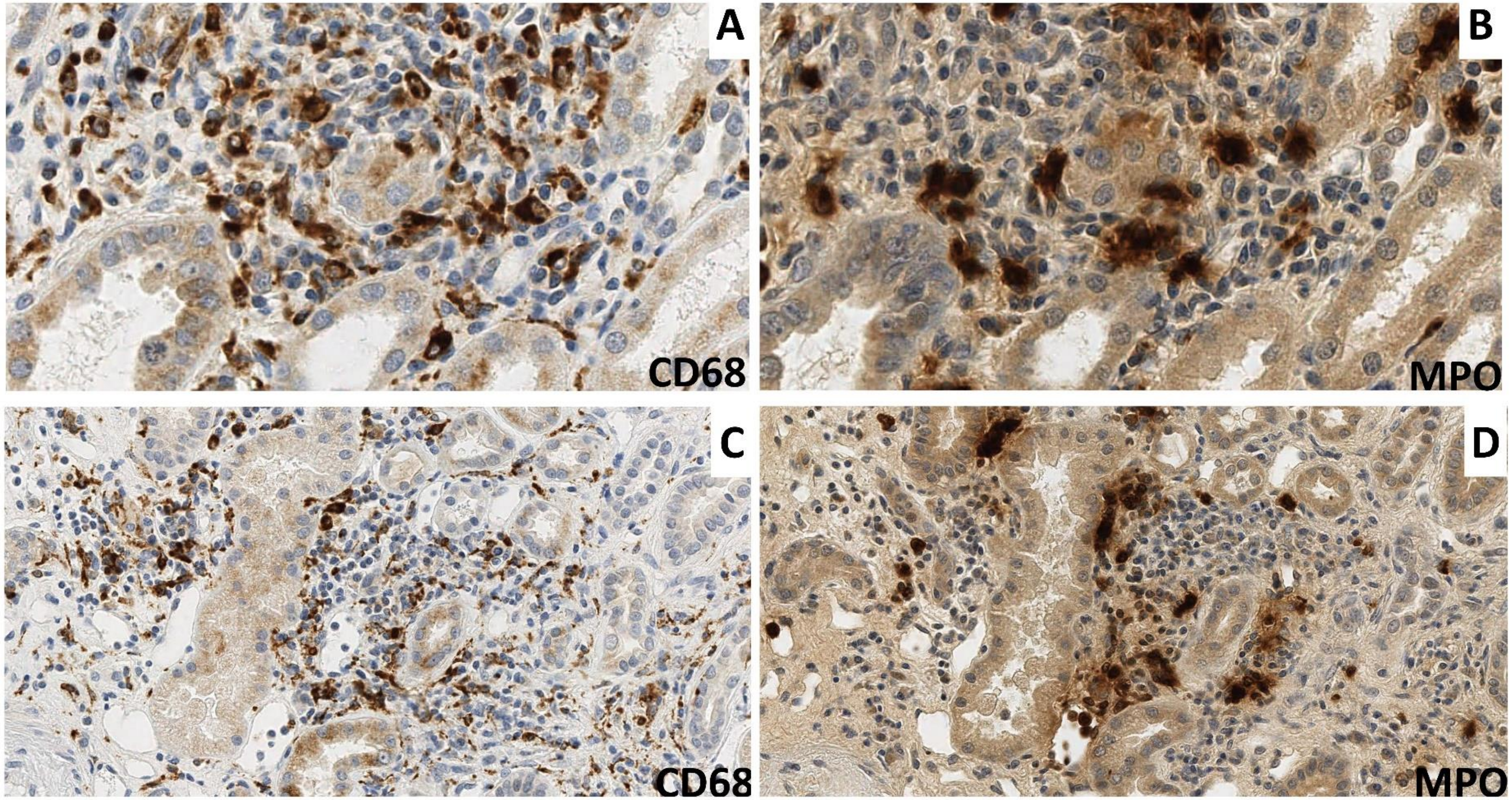

Supplementary Figure Legend

Representative CD68 (A 40X, C 20X) and MPO (B 40X, D 20X) immunostaining.

Supplementary tables

Supplementary Table 1: Evolution at last follow-up

patient #	Intial sCreatinine (µmol/l)	Initial eGFR (ml/min/1.73m²)	Initial Proteinuria (g/day)	Follow-up post KB (months)	Last FU CKD stage	Last FU ESRD	Last FU sCreatinine (µmol/l)	Last FU eGFR (ml/min/1.73m²)	Last FU Proteinuria (g/day)	Last FU Renal response*	Steroids	Vidaza	JAK Inhibitors	other	Infectious complications	death
1	70	110	0.12	90	1	0	90	77	0.5	1	1	0	0	anti-IL17	Pneumonias, osteitis, multiple sepsis	1
2	1300	0	1.8	17	4	0	281	18	NA	2	1	0	1	Anti-IL1	Pneumonias, pyelonephritis	0
3	169	35	0.7	32	3	0	116	54	NA	2	1	0	1	MTX	Colitis with perforation	0
4	72	90	5.6	25	2	0	90	77	0	2	1	1	1	anti-IL6R, anti-TNF	Pneumonias, orchiepididymitis	0
5	980	NA	6.3	27	2	0	77	85	NA	2	1	1	1	anti-IL6R	0	0
6	43	113	3.0	5	1	0	65	95	0.8	2	1	0	0	MTX, LEN, MMF	0	1
7	296	NA	1.2	41	4	0	244	21	0.9	1	0	0	1		0	NA
8	191	29	4.3	0	4	0	214	29	4.3	NA	1	0	1	0	0	0
9	239	22	0.3	0	4	0	228	22	0.3	1	1	0	1	Colchicine, Anti-IL1, Anti-IL6R	Pneumonias, herpes zoster, COVID19	0
10	427	12	9.0	12	5	1	NA	NA	NA	0	1	0	0	Anti-IL1	0	0
11	207	26	3.8	17	3	0	115	52	0	2	1	0	0	0	COVID19	0

***Renal response :** *(eGFR> 30% and resolution of Proteinuria : 2; stable eGFR+/- 10%, no Proteinuria: 1; otherwise : 0)*

CKD : Chronic Kidney Disease; ESRD : End Stage Renal Disease; KB : Kidney Biopsy; LEN : Lenalidomid; MMF : Mycophenolate Mofetil; MTX : Methotrexate

Supplementary Table 2: Histological, immunohistochemical and molecular characteristics

patient #	Main histological diagnosis	Type of *UBA1* mutation	i	ti	IFTA	i-IFTA	Interstitial mononuclear cells	Interstitial polymorphonuclear cells	MPO	CD68	CD3	CD15	IF	%VAF and depth of coverage (Kidney)	Others variants
1	IgAN	*c.122T>C p.Met41Thr*	0	0	10%	0	NA	NA	NA	NA	NA	NA	Mesangial IgA	ND	ND
2	AIN	*c.118-1G>C Splice mutation*	1	3	50%	3	+++	++	NC	+++	+++	+	0	ND	ND
3	AIN	*c.121A>C p.Met41Leu*	0	2	60%	3	+++	+	+++	+++	NA	NA	0	ND	ND
4	AIN+MCD	*c.122T>C p.Met41Thr*	0	1	30%	2	+++	0-+	++	++	+++	+	0	2% (750x)	None
5	ANCA - PICGN	*c.121A>G p.Met41Val*	2	2	0	0	+++	+++	+	++	+	+	0	17% (902x)	None
6	MCD (w/ IgA deposits)	*c.118-1G>C Splice mutation*	0	0	10%	0	+	0	++	++	++	0-+	Mesangial IgA	0% (510x)	None
7	Acute tubular necrosis, mesangial expansion	*c.122T>C p.Met41Thr*	0	0	70%	1	+++	0-+	+	+++	++	+	Rare Mesangial IgM and C3, no light chain deposits	3% (639x)	*SF3B1, ZRSR2**
8	Vascular and diabetic	*c.121A>C p.Met41Leu*	0	1	80%	1	+++	0-+	++	++	++	0-+	No glomeruli	8% (993x)	None
9	Acute tubular necrosis, mesangial expansion	*c.122T>C p.Met41Thr*	0	0	10%	2	+	0	NA	NA	NA	NA	0	4% (933x)	None
10	AA amyloidosis	*c.122T>C p.Met41Thr*	0	1	90%	1	+	0	NA	NA	NA	NA	0	7% (640x)	None
11	AIN	*c.122T>C p.Met41Thr*	1	1	10%	1	+	0	0-+	++	NA	NA	C3 deposits (vessels)	4% (632x)	*DNMT3A, NFE2***

IgAN : IgA Nephropathy; AIN : Acute interstitial nephritis; IF: Immunofluorescence; MCD : Minimal change disease; PICGN : Pauci-immune crescentic glomerulonephritis; i : inflammation in non-fibrotic areas; ti : total inflammation; IFTA : % of interstitial fibrosis and tubular atrophy; i-IFTA : inflammation in fibrotic areas, NA : non-available; NC : Non contributive. ND : Not Done

Quantification of Banff criteria: 0: absent; 1: mild; 2: moderate; 3: severe

Quantification of interstitial mononuclear cells, polymorphonuclear cells, MPO, CD68, CD3, CD15 expression in interstitial inflammatory cells:

0: absent; +: mild (<10% cells); ++ moderate (10-25% cells); +++: severe (>25%)

IF: Immunofluorescence: IF study included IgG, IgA, IgM, C3 C1q, kappa light chain, lambda light chain

*SF3B1 c.2242A>G p.Lys748Glu : VAF =1% (1393x) ; ZRSR2 c.112dupA p.Arg38Lysfs*17 : VAF = 2% (731x)

**DNMT3A c.1903C>T p.Arg635Trp : VAF = 6% (815x) ; NFE2 c.758dup p.Leu254Ilefs*51 : VAF = 1% (1634

Supplementary Table 3: Detailed histological description

Patient number	Main Diagnosis	Glomeruli		Tubulo-interstitial lesions	Vascular lesions		
		Number	Number of sclerotic glomerulus	Acute tubular necrosis	v	cv	ah
1	IgAN	12	2	0	0	1	0
2	AIN	10	2	2	0	2	0
3	AIN	22	2	0	0	2	0
4	AIN+MCD	30	2	0	1	2	1
5	ANCA - PICGN	13	0	2	0	2	0
6	MCD (w/ IgA deposits)	9	0	1	0	1	0
7	Acute tubular necrosis, mesangial expansion	8	3	2	0	2	1
8	Vascular and diabetic	15	7	0	0	3	3
9	Acute tubular necrosis, mesangial expansion	NA	NA	2	0	2	2
10	AA amyloidosis	NA	NA	1	0	1	1
11	AIN	11	1	0	0	2	0

IgAN : IgA Nephropathy; AIN : Acute interstitial nephritis; MCD : Minimal change disease; PICGN : Pauci-immune crescentic glomerulonephritis; v : intimal arteritis, cv : arterial fibrous intimal thickening, ah = arteriolar hyalinosis , NA : non-available Quantification: 0: absent; 1: mild; 2: moderate; 3: severe

STROBE Statement—checklist of items that should be included in reports of observational studies

	Item No.	Recommendation	Page No.	Relevant text from manuscript
**Title and abstract**	1	(*a*) Indicate the study's design with a commonly used term in the title or the abstract	2	
		(*b*) Provide in the abstract an informative and balanced summary of what was done and what was found	NA	
**Introduction**				
Background/rationale	2	Explain the scientific background and rationale for the investigation being reported	2	
Objectives	3	State specific objectives, including any prespecified hypotheses	2	
**Methods**				
Study design	4	Present key elements of study design early in the paper	2	
Setting	5	Describe the setting, locations, and relevant dates, including periods of recruitment, exposure, follow-up, and data collection	2 and supplementary material	
Participants	6	(*a*) *Cohort study*—Give the eligibility criteria, and the sources and methods of selection of participants. Describe methods of follow-up<br>*Case-control study*—Give the eligibility criteria, and the sources and methods of case ascertainment and control selection. Give the rationale for the choice of cases and controls<br>*Cross-sectional study*—Give the eligibility criteria, and the sources and methods of selection of participants	2	
		(*b*) *Cohort study*—For matched studies, give matching criteria and number of exposed and unexposed<br>*Case-control study*—For matched studies, give matching criteria and the number of controls per case		

Variables	7	Clearly define all outcomes, exposures, predictors, potential confounders, and effect modifiers. Give diagnostic criteria, if applicable	supplementary material
Data sources/ measurement	8*	For each variable of interest, give sources of data and details of methods of assessment (measurement). Describe comparability of assessment methods if there is more than one group	*2*
Bias	9	Describe any efforts to address potential sources of bias	3
Study size	10	Explain how the study size was arrived at	3

Continued on next page

Quantitative variables	11	Explain how quantitative variables were handled in the analyses. If applicable, describe which groupings were chosen and why	NA
Statistical methods	12	(*a*) Describe all statistical methods, including those used to control for confounding	2 and supplementary material
		(*b*) Describe any methods used to examine subgroups and interactions	
		(*c*) Explain how missing data were addressed	
		(*d*) *Cohort study*—If applicable, explain how loss to follow-up was addressed<br>*Case-control study*—If applicable, explain how matching of cases and controls was addressed<br>*Cross-sectional study*—If applicable, describe analytical methods taking account of sampling strategy	
		(*e*) Describe any sensitivity analyses	
**Results**			
Participants	13*	(a) Report numbers of individuals at each stage of study—eg numbers potentially eligible, examined for eligibility, confirmed eligible, included in the study, completing follow-up, and analysed	3
		(b) Give reasons for non-participation at each stage	
		(c) Consider use of a flow diagram	
Descriptive data	14*	(a) Give characteristics of study participants (eg demographic, clinical, social) and information on exposures and potential confounders	3
		(b) Indicate number of participants with missing data for each variable of interest	
		(c) *Cohort study*—Summarise follow-up time (eg, average and total amount)	
Outcome data	15*	*Cohort study*—Report numbers of outcome events or summary measures over time	*3*

		Case-control study—Report numbers in each exposure category, or summary measures of exposure	*3*
		Cross-sectional study—Report numbers of outcome events or summary measures	
Main results	16	(*a*) Give unadjusted estimates and, if applicable, confounder-adjusted estimates and their precision (eg, 95% confidence interval). Make clear which confounders were adjusted for and why they were included	3
		(*b*) Report category boundaries when continuous variables were categorized	
		(*c*) If relevant, consider translating estimates of relative risk into absolute risk for a meaningful time period	

Continued on next page

Other analyses	17	Report other analyses done—eg analyses of subgroups and interactions, and sensitivity analyses	
**Discussion**			
Key results	18	Summarise key results with reference to study objectives	4
Limitations	19	Discuss limitations of the study, taking into account sources of potential bias or imprecision. Discuss both direction and magnitude of any potential bias	5
Interpretation	20	Give a cautious overall interpretation of results considering objectives, limitations, multiplicity of analyses, results from similar studies, and other relevant evidence	4
Generalisability	21	Discuss the generalisability (external validity) of the study results	4
**Other information**			
Funding	22	Give the source of funding and the role of the funders for the present study and, if applicable, for the original study on which the present article is based	5

*Give information separately for cases and controls in case-control studies and, if applicable, for exposed and unexposed groups in cohort and cross-sectional studies.

Note: An Explanation and Elaboration article discusses each checklist item and gives methodological background and published examples of transparent reporting. The STROBE checklist is best used in conjunction with this article (freely available on the Web sites of PLoS Medicine at http://www.plosmedicine.org/, Annals of Internal Medicine at http://www.annals.org/, and Epidemiology at http://www.epidem.com/). Information on the STROBE Initiative is available at www.strobe-statement.org.
